# ATP release mediated by pannexin-3 is required for plasma cell survival via P2X4 receptors in bone marrow

**DOI:** 10.1007/s11302-024-10024-z

**Published:** 2024-05-21

**Authors:** Sonia Paz-López

**Affiliations:** Biomedical Research Centre, School of Biological Sciences, University of East Anglia, Norwich, NR4 7TJ UK

**Keywords:** ATP release, Bone marrow, Plasma cell, P2X4 receptor, Panx3

## Article summary

Extracellular adenosine-5′-triphosphate (ATP) is an important extracellular messenger that influences cellular processes, such as proliferation, apoptosis, and differentiation. Once ATP is released into the extracellular space, it can bind purinergic receptors in the cells that released the nucleotide or in neighboring cells. In a recent publication in *Nature*, Ishikawa et al. [1] showed that extracellular ATP released by osteoblasts via pannexin-3 channels in gap junctions in the bone marrow niche cells acts in a paracrine fashion, binding P2X4 receptors in plasma cells. The expression of both channels is required for appropriate antibody production and plasma cell survival. In contrast, genetic deletion of either Panx3 in osteoblastic cells or P2X4 receptors in plasma cells caused plasma cell depletion and reduced their ability to produce antibodies in vitro. In addition, treatment with a selective P2X4 receptor antagonist, 5-BDBD, in vitro and in vivo reduced serum antibody titer and plasma cell survival. The authors also illustrate the potential implication of this mechanism in an autoimmune disease, such as systemic lupus erythematosus, as P2X4 receptor activity in bone marrow plasma cells is shown to inhibit endoplasmic reticulum-induced apoptosis.

## Commentary

Bone marrow is a squishy tissue located in the center of most bones and is highly innervated. It is the primary niche of hematopoietic stem cells, from which red blood cells, platelets, and white blood cells (plasma cells, granulocytes, T lymphocytes, etc.) are derived. Therefore, the primary role of hematopoietic stem cells is supporting an appropriate blood cell turnover and supporting the immune system. However, these are not the only stem cells in the tissue, as mesenchymal stem cells are also present. Bone and cartilage regenerating cell types, such as osteoblast and chondrocytes, are formed by differentiating the mesenchymal stem cells [2].

Ishikawa et al. focused on the role of P2X4 receptors in supporting the survival and functionality of bone marrow plasma cells, which are immune cells that produce large amounts of antibodies and contribute to long-term immunity. This cell type is responsible for the body’s primary immunoglobulin (Ig) production [3]. Accordingly, this work used the measurements of three main Igs (IgG, IgM, IgA) to assess plasma cell function and survival in different experimental settings. Conversely, osteoblasts are bone-forming cells that can release ATP under mechanical stimulation, infection, or apoptotic processes [4]. In addition, and relevant to its recent publication, previous work by Ishikawa in 2011 [5] showed that osteoblasts express the pannexin channel 3 (Panx3) at the mRNA and protein levels. Together with many other studies, they demonstrated that this hemichannel is a mediator of ATP release in different cell beds [5–8]. Once released, ATP is proposed to bind P2X4 receptors expressed in bone marrow plasma cells. P2X4 receptors are one of the seven ionotropic receptors of the P2X receptor subfamily. Nucleotide receptors are subdivided into two subfamilies named P2X and P2Y receptors [9]. Their sequence homology, topology, and pharmacological profile decided their classification. P2X receptors are ionotropic, while P2Y receptors are metabotropic, sharing their G protein–coupled receptor nature with P1 receptors (nucleoside receptors) [10, 11]. ATP is the preferred agonist of the P2X receptors [12], with EC_50_ values at the P2X4 receptor in the range of 1 to 10 µM [13]. P2X4 receptors also demonstrate slow desensitization. When studying the expression of P2X and P2Y receptors at the RNA level in mouse bone marrow plasma cells, the P2X4 receptor had the highest expression, followed by P2Y_14_ receptors. However, the preferred agonists at P2Y_14_ receptors are UDP and UDP-glucose [14]. Note that the p2Y_10_ receptor was included in this mRNA expression data; this G protein–coupled receptor (GPCR) is not a nucleotide receptor of the purinergic P2Y family, but a lysophospholipid receptor that is believed to be an orphan GPCR [15]. Based on previous studies on the function and ATP release of the Panx3 channel in osteoblasts and the commented expression data, Ishikawa et al. proposed that P2X4 receptors in plasma cells of this very characteristic niche are the primary sensor of ATP.

Using Panx3 knockout (KO) mice, the authors determined the impact of this channel-dependent activity on the production of IgA, IgG, and IgM in sera (the liquid fraction of blood) and the frequency of plasma cells in the bone marrow compared to other antibody-secreting cells in hematopoietic organs (ex. spleen). They demonstrated that the absence of the hemichannel abolished the production of plasma cells in bone marrow, but not in the other cell niches. Consistently, Ig levels significantly decreased in the KO mice, and organ specificity was also conserved. An important note is that the number of plasma cells was determined using flow cytometry. However, the authors also indirectly quantified the number of viable plasma cells by measuring the production of Ig. To study whether osteoblastic cells support the function of bone marrow plasma cells, pre-induced osteoblastic cells (from calvarial cells, skull bone) of WT and Panx3 KO mice were established and co-cultured with plasma cells of both mice models. After 4 days in culture without osteoblasts, bone marrow cells from WT mice lost their capacity to produce IgG. However, this production was rescued when co-cultured with osteoblast Panx3-positive cells, and this effect was not the case in the presence of Panx3-negative osteoblastic cells, illustrating the modulation of plasma cells by the bone-forming cells. Once the Panx3 osteoblastic dependency of plasma cells was demonstrated, the following step was to show that it was directly linked to purinergic signaling. Extracellular ATP released was quantified and found to be significantly higher in osteoblastic Panx3-positive cells than in the negative cultures. In addition, when treated with ATP, bone marrow plasma cells from the WT mice showed recovered capacity of secreting IgG, which was not the case for other nucleotides (ADP and AMP), making this process ATP-dependent. Consequently, the authors focused on P2X4 receptors, rather than P2Y_14_ receptors, whose preferred agonist is a different nucleotide.

The next step was determining whether P2X4 receptors were required for the activity of bone marrow plasma cells. They consistently and systematically replicated the experimental settings previously performed for Panx3 WT and KO mice, now using WT and conditional P2X4 receptor KO mice (Cre/loxP technology). The absence of P2X4 receptors abolished the production of plasma cells in the bone marrow and reduced the Ig levels, mirroring the results of the Panx3 KO mice. Both observed effects were also organ-specific. Furthermore, when treated with ATP, bone marrow plasma cells from the WT mice depended on ATP to secrete IgG. In contrast, the P2X4 receptor KO cells could not produce Ig in the presence or absence of the nucleotide. The authors complemented these data using a different mutagenesis setting to temporally induce P2X4 receptor gene deletion in mice (tamoxifen-induced Cre-recombinase technology). This strategy was as valuable as the previous P2X4 receptor KO mice model, and the results were again conserved and organ-specific as previously described for the other model.

The authors also considered the pharmacological inhibition of P2X4 receptor. First, they used two broad-spectrum antagonists, suramin and PPADS, to study their impact on mouse viable bone marrow cells. Mouse P2X4 receptors (mP2X4) are PPADS-sensitive, with a half-maximal inhibitory concentration (IC_50_) of 10 µM, but are less sensitive to suramin [16]. Both reduced the number of viable bone marrow cells. Next, 5-BDBD, a selective P2X4 receptor antagonist, was used and was found to abolish the antibody-secreting cells. In contrast, a selective P2 × 7 receptor antagonist had no effect. Since some of the subsequent experimental results are established on a systemic lupus erythematosus mouse model and the P2 × 7 receptor has been demonstrated to be involved in the disease, this analysis excludes P2 × 7 receptor involvement in the processes. Wild-type mice were also treated with 5-BDBD at concentrations previous studies have used before [17, 18], and the number of viable bone marrow plasma cells was significantly decreased, as previously reported for the broad-spectrum antagonists. The effect was also localized to the bone marrow cell niche. To complement these data and dig further into the role of P2X4 receptors on Ig production by plasma cells, Ishikawa and colleagues induced NP-KLH/alum immunization in WT and Panx3 mice, then exposed them to 5-BDBD and tracked the changes of antibody production. This also showed a 5-BDBD-dependent reduction in both IgM and IgG antibody titers, which persisted once the treatment was stopped.

As noted above, this study also investigated P2X4 receptors in bone marrow plasma cells as a possible target to modulate pathogenic antibody production in two systemic lupus erythematosus models (SLE) (NZB/W mice and induced WT mice to produce anti-dsDNA antibodies and proteinuria). SLE key severity indicators are the production of antibodies against double-stranded DNA and the development of proteinuria and albuminuria [19]. Their results revealed a powerful impact of all three quantified parameters when the animals were treated with the selective P2X4 receptor antagonist in both models. Furthermore, progressive proteinuria and high levels of anti-double-stranded DNA develop into kidney damage. Consistently with the previous results described, kidney samples of the 5-BDBD-exposed animals at the latest time showed improved histological conditions, with recovered pigmentation and lower tubular hypertrophy.

Given that some strategies to eliminate unfit plasma cells involve inducing endoplasmic reticulum (ER) stress apoptosis, the possibility of P2X4 receptors contributing to ER homeostasis was investigated by studying the ATF4–CHOP pathway, which is part of the apoptosis-leading event upon ER acute stress. Inhibition of P2X4 receptor by 5-BDBD evoked increased levels of ER stress-associated factors, including ATF4. Knocking out Chop, a pro-apoptotic target of ATF4, prevented the antagonist from modulating the function of bone marrow plasma cells, illustrating the dependency on Chop in P2X4 receptor-induced plasma cell depletion.

Therefore, this research shows that P2X4 receptors support ER homeostasis and cell survival in bone marrow plasma cells through a mechanism dependent on sensing ATP release from osteoblasts. In addition, these results indicate that P2X4 receptor inhibition could be used to deplete the production of pathogenic antibody levels in SLE and potentially in other pathological conditions.

## Data Availability

Data is provided within the manuscript.
